# Entomopathogenic Nematodes Combined with Adjuvants Presents a New Potential Biological Control Method for Managing the Wheat Stem Sawfly, *Cephus cinctus* (Hymenoptera: Cephidae)

**DOI:** 10.1371/journal.pone.0169022

**Published:** 2016-12-22

**Authors:** Scott L. Portman, Sindhu M. Krishnankutty, Gadi V. P. Reddy

**Affiliations:** 1 Montana State University, Western Triangle Agricultural Research Center, Conrad, MT, United States of America; 2 Xavier University/USDA-APHIS PPQ, Buzzards Bay, MA, United States of America; University of Idaho, UNITED STATES

## Abstract

The wheat stem sawfly, (*Cephus cinctus* Norton) Hymenoptera: Cephidae, has been a major pest of winter wheat and barley in the northern Great Plains for more than 100 years. The insect’s cryptic nature and lack of safe chemical control options make the wheat stem sawfly (WSS) difficult to manage; thus, biological control offers the best hope for sustainable management of WSS. Entomopathogenic nematodes (EPNs) have been used successfully against other above-ground insect pests, and adding adjuvants to sprays containing EPNs has been shown to improve their effectiveness. We tested the hypothesis that adding chemical adjuvants to sprays containing EPNs will increase the ability of EPNs to enter wheat stems and kill diapausing WSS larvae. This is the first study to test the ability of EPNs to infect the WSS, *C*. *cinctus*, and test EPNs combined with adjuvants against *C*. *cinctus* in both the laboratory and the field. Infection assays showed that three different species of EPNs caused 60–100% mortality to WSS larvae. Adding Penterra, Silwet L-77, Sunspray 11N, or Syl-Tac to solutions containing EPNs resulted in higher WSS mortality than solutions made with water alone. Field tests showed that sprays containing *S*. *feltiae* added to 0.1% Penterra increased WSS mortality up to 29.1%. These results indicate a novel control method for WSS, and represent a significant advancement in the biological control of this persistent insect pest.

## Introduction

The wheat stem sawfly, *Cephus cinctus* Norton (Hymenoptera: Cephidae), has been an important pest of cereal crops in the northwest region of North America for more than 100 years [1, 2]. The wheat stem sawfly (WSS) attacks mostly winter wheat (*Triticum aestivum* L.), but is also known to damage barley (*Hordeum vulgare* L.) and rye (*Secale cereale* L.) [3, 4]. Yield losses caused by the WSS are most prevalent in the northern Great Plains, including Montana, North Dakota, South Dakota, Minnesota, Saskatchewan, Alberta, and Manitoba [5, 6]. Infestation levels of more than 70% have been reported [7] and economic loss from crop damage caused by this insect has been estimated at $250 million USD per year in the state of Montana alone [8].

Much of the WSS’s resiliency is linked to the insect’s biology and cryptic nature. Short-lived females (7–10 days) use their saw-like ovipositor to insert their eggs inside the elongating stems of host plants [5, 4]. Once the larvae hatch, they live as stem borers, feeding on the parenchyma and vascular tissues of the stems [5]. More than one egg can be deposited inside a stem, but due to conspecific competition, only a single larva typically survives [9, 10] Mature larvae move to the basal sections of their stems and chew a notch around the inside of the stems, weakening the stalks [11]. Below the notch, the larvae construct a plug from frass and plant particles to close up the exposed stem lumen when the weakened stalks break off and fall away [4]. The remaining wheat “stubs” function as hibernaculums where the larvae pass the winter months in a state of obligatory diapause [5, 4]. WSS causes severe crop loss because infested wheat plants produce lower quality kernels and fallen stems cannot be gathered by combine harvester machines [6].

Management of WSS is unusually challenging because the larvae and pupae reside inside the stems, which provide protection from contact insecticides [5, 12]. Recently the State of Montana approved Thimet^®^ 20-G (Amvac Chemical Corporation, Los Angeles CA), a powerful systemic organophosphate, for control of WSS [13]. Although Thimet is effective at killing WSS [14, 15], this chemical is costly and poses many health and environmental risks [16, 17, 18]. In addition, agronomic control strategies, such as the development of resistant (solid stem) wheat varieties and tillage practices that attempt to destroy diapausing larvae, have not succeeded in reducing WSS populations below economic threshold [5, 4], and solid stem wheat varieties are reported to produce lower yields than hollow stem varieties, which adds the pest’s economic impact [19]. Therefore, winter wheat and barley growers in the Golden Triangle of Montana have expressed considerable interest in the development of low-risk control strategies (e.g. biological control) which can achieve economical and sustainable management of WSS.

Two parasitoid wasps (*Bracon cephi*, Gahan and *Bracon lissogaster*, Muesebeck; Hymenoptera, Braconidae) are found associated with WSS in the Golden Triangle; however, these parasitoids are not providing sufficient control because local levels of WSS parasitism are highly variable [20]. In addition, these parasitoids are difficult to rear and mass releases have not been effective at establishing large populations throughout the region [20, 21]. Entomopathogenic nematodes are another biological control option that could be used in conjunction with parasitoids and other control strategies to improve management of WSS. Entomopathogenic nematodes (EPNs) are soil-dwelling round worms (Phylum: Nematoda, Order: Rhabditida) that specialize in parasitizing insects. EPN infective juveniles (IJs) enter the insect host and release symbiotic bacteria, resulting in septicemia that kills the insect 24–48hrs later. EPN juveniles feed on the mix of bacteria and liquefied insect tissue, mature, and reproduce inside the host. When the insect’s nutrient resources have been exhausted, a new generation of IJs exit the carcass in search of new hosts [22].

EPNs have been used successfully to manage a large number of insect pests, including some insects that live above-ground and stem borers [23, 24, 25, 26, 27, 28]. EPNs are generally applied to above ground vegetation using conventional spray equipment, but EPNs will only survive for a few hours on exposed foliage because they desiccate quickly and they are sensitive to UV rays [29, 22]. However, adding EPNs to solutions containing adjuvants (e.g. surfactants, wetting agents, oils) or humectants (e.g. Barricade^®^ fire gel) has been shown to improve their control efficiency against above-ground and foliar insects [30, 31, 32, 33, 27, 28]. This suggests that EPNs might be effective against a wider range of foliar insect pests when applied in conjunction with chemical additives that help to prolong their survival above ground.

Despite their efficacy at controlling other above-ground insect pests, the use of EPNs against WSS is largely unexplored. One recent study showed a significant reduction in the number of WSS infested wheat stems collected from plots treated with sprays containing EPNs [34]. Although this study showed a correlation between EPN treatment and WSS reduction, EPN infections of the diapausing larvae were never verified. One obstacle to EPN infection of WSS is the hydrophobic plug formed by the larvae prior to diapause [4]. The plug is porous, but its hydrophobicity prevents water from readily absorbing into the plug. Chemical adjuvants can decrease the surface tension of a liquid and increase the liquid’s dispersion properties and rate of absorbance into a hydrophobic matrix [35]. Spray mixtures which contain adjuvants that absorb into plugs quickly could allow EPNs to penetrate the plugs and infect the larvae inside the stems. Some adjuvants, such as the polyacrylate gel Barricade^®^, improve adhesion of spray droplets and provide a protective medium to EPNs [31, 27, 28]. Here we verify that EPNs possess the ability to infect and kill WSS larvae; then we tested the hypothesis, in the laboratory and the field, that treating wheat stubble with EPN solutions containing adjuvants will result in higher WSS mortality compared to EPN treatments mixed with water alone.

## Materials and Methods

### EPN infection assay

To determine if WSS was susceptible to EPN infection, diapausing WSS larvae were exposed to three species of EPNs: *Heterorhabditis indica*, *Steinernema kraussei*, *and Steinernema feltiae*. Wheat stubble containing overwintering WSS was collected from a harvested Judee winter wheat field in Teton County, Montana (N47° 52.1916’ W112° 35.5956’). Permission to collect wheat stubble samples was granted by local private landowners: James Bjelland (Podera county, MT), Ken Johnson (Podera county, MT) and Dan Schuler (Teton county, MT). The research activities reported here did not involve, pose a risk to, or harm any endangered or protected species. Using a scalpel, wheat stems were sliced open along the long axis and larvae were gently removed with forceps or a dissecting needle. Care was taken not to injure the larvae during removal and all larvae were inspected under a stereomicroscope to ensure they had no prior injuries that could affect their mortality or susceptibility to infection by EPNs. EPNs were obtained from Becker Underwood Inc. (now BASF Corp., Ames IA) and stored at 4°C.

Seventy-five WSS larvae were placed singularly in 55mm plastic Petri dishes (Bioplast Manufacturing L.L.C., Bristol, PA) containing two pieces of moistened 55mm Whatman^®^ filter paper (GE Healthcare Bio-Sciences, Malborough, MA). To test different concentrations of infective juveniles (IJs) against WSS, IJs from each EPN species were added to distilled water at concentrations of 200, 400, 800 and 2000 IJs/ml. Using a pipette, EPNs were applied by placing a 25ul droplet of EPN solution onto the filter paper next to the WWS larva–EPN application rates were 50, 100, 200, and 500 IJ/larva. Five WSS larvae were treated with each EPN solution (3 EPNs × 4 concentrations × 5 larvae). Applications using 25ul of distilled water without nematodes served as negative controls. After treatment, Petri dishes were sealed with Parafilm M^®^ (Bemis Company Inc., Neenah, WI) and moved to a 25°C incubator.

Larval mortality was assessed every day, for three days following EPN applications. Dead larvae were immediately moved to fresh Petri dishes lined with moist filter paper. EPN infected WSS larvae rapidly turn reddish-brown in color; thus, they can be easily distinguished from uninfected larvae. EPN infections were confirmed using the “white trap” method [36]. After 7 days, all white traps were evaluated for the presence of IJs under a stereomicroscope. Following mortality assessments, the experiment was repeated (N = 2) to confirm the results. Daily percent mortalities were averaged within treatments to obtain mean larval percent mortalities two and three days after EPN exposure.

### Adjuvant absorbance assay

To test the ability of different chemical solutions to absorb into the hydrophobic plugs, we made artificial plugs from natural plug material and measured the rate of absorbance for each solution. Artificial plugs were used because there is a large amount of variability in the size of natural plugs (0.2–1.0 mg) and natural plugs are extremely fragile and crumble easily during removal. Wheat stubble containing WSS larvae were collected from two harvested Judee winter wheat fields in Pondera county MT (N48°10.567’ W111°32.872’; N48°11.397’ W111°25.843’) and one in Teton county MT (N47°52.360’ W111°40.324’). Dirt and debris were removed from each stem and clean stems were kept in 473 ml plastic deli containers; deli containers with stems were stored in an incubator at 8°C. To create the artificial plugs, ~200 natural plugs were removed from the wheat stubble and ground into a powder of uniform consistency. Plug material was slightly moistened with distilled water and the open ends of Wilmad-Lab Glass^®^ capillary tubes, which approximated the size of a wheat stem (2.2 mm ID, 2.5 mm OD; SP Industries Inc., Warminster, PA), were gently pushed into the moistened plug material. Artificial plugs were allowed to dry overnight inside the capillary tubes; plugs were removed from the tubes the following day. Artificial plugs were 4–5 mm in length and weighed an average of 3.1 mg.

Nine commercial adjuvants (Adigor^®^, Advantage^®^, Alypso^®^, Penterra^®^, R-11^®^, Silwet L-77^®^, Sun Ag Oil^®^, Sunspray 11N^®^, and Syl-Tac^®^) were mixed according to the manufacturers’ recommendations; Barricade (Barricade International Inc, Hobe Sound, FL), Tween 80^®^, Triton X-100^®^, and Urea (Thermo Fisher Scientific, Waltham, MA) were mixed at concentrations of 1.0%, 1.0%, 1.0% and 5.0% respectively (Table 1). Because Sun Ag Oil and Sunspray 11N contain mostly mineral oil, which does not readily dissolve in water, 0.05% Triton X-100 was added to both as an emulsifier. 5.0 ml of each solution was poured into 55 mm glass petri dishes–distilled water served as the control. Artificial plugs were released singularly into each solution and a stop watch recorded the time (seconds) required for the plugs to become completely saturated–recording did not continue past 300 sec. The assay was performed three times (N = 3) for each solution (Table 2) and absorbance times were averaged to obtain mean saturation times.

**Table 1:** Adjuvant: product name, manufacturer, main chemical ingredients, and formulation.

Product Name	Manufacturer	Chemical Ingredients	Adjuvant Added	Volume H_2_O (ml)	Solution Conc. (%)
Adigor	Syngenta Crop Protection, LLC.	fatty alcohol alkoxylate	0.5 ml	99.5	0.5
Advantage	Wilbur-Ellis Co.	ammonium alky ether sulfate	0.78 ml	99.22	0.8
Alypso	Precision Laboratories, LLC.	alkyl polyglucoside ester	0.31 ml	99.69	0.3
Barricade	Barricade International, Inc.	sodium polyacrylate + modified vegetable oil	1.0 ml	99.0	1.0
Penterra	Geoponics, Inc.	propylene glycol	0.13 ml	99.87	0.1
R-11	Wilbur-Ellis Co.	alkylphenol ethoxylate, butyl alcohol, dimethylpolysiloxane	0.78 ml	99.22	0.8
Silwet L-77	Helena Chemical Co.	siloxane polyalkyleneoxide copolymer	0.1 ml	99.9	0.1
Sun Ag Oil	HollyFrontier Refining, LLC.	mineral oil + additives (50–100 light, 0–50 heavy)	1.0 ml	99.0	1.0
Sunspray 11N	HollyFrontier Refining, LLC.	mineral oil + additives (20–30 light, 70–80 heavy)	1.0 ml	99.0	1.0
Syl-Tac	Wilbur-Ellis Co.	modified vegetable oil + silicone polymer	0.39 ml	99.61	0.4
Triton X-100	Thermo Fisher Scientific, Inc.	polyethylene oxide polymer	1.0 ml	99.0	1.0
Tween 80	Thermo Fisher Scientific, Inc.	polyethylene glycol sorbitan monooleate	1.0 ml	99.0	1.0
Urea	Thermo Fisher Scientific, Inc.	carbamide	5.0 g	100	5.0

**Table 2:** Number of seconds required for three artificial plugs (avg. length: 4–5 mm; avg. mass: 3.1 mg) to become completely saturated when placed in 5.0 ml of carrier solution. Recordings were stopped after 300 seconds had elapsed.

Solution	Saturation Time (Sec)		
	Trial 1	Trial 2	Trial 3
Adigor	7.4	20.6	9.2
Advantage	>300	>300	>300
Alypso	129.7	117.4	148.5
Barricade	>300	>300	>300
Distilled H_2_O	>300	>300	>300
Penterra	14.4	13.1	11.3
R-11	4.1	4.2	4.2
Silwet L-77	24.6	14.2	27.7
Sun Ag Oil	56.6	79.8	72.5
Sunspray 11N	44.3	70.3	52.1
Syl-Tac	6.3	4.9	8.3
Triton X-100	>300	>300	>300
Tween 80	>300	276	>300
Urea	>300	>300	>300

### Laboratory assay of EPNs with carrier solutions

To determine if EPN solutions containing different chemical additives would allow EPNs to pass through the plug formed by the WSS and come into contact with the insect, we applied carrier solutions containing EPNs to the tops of wheat stubs. Although *H*. *indica* was previously found to cause high mortality in WSS larvae (Table 3), *H*. *indica* was not used for further testing because this species prefers warm moist environments and is generally only found in tropical or subtropical climates (22). *H*. *indica* was replaced with *S*. *riobrave* because this species survives in dryer climates–such as the semi-arid climate of the northern Great Plains. Pilot trials tested six species of EPNs (*H*. *bacteriophora*, *S*. *carpocapsae*, *S*. *feltiae*, *Steinernema glaseri*, *S*. *kraussei*, *and Steinernema riobrave*). However, only *H*. *bacteriophora*, *S*. *feltiae*, *and S*. *riobrave* produced significant mortality (>30%), thus, subsequent trials only included these three species. All species of EPNs used in this experiment were commercially available and included both cruisers and ambushers [22]. Commercial availability of an EPN was an important selection criterion because we wanted to test only species that growers could readily obtain in large numbers.

**Table 3:** Average (mean ± SE) percent mortality (N = 5) of wheat stem sawfly larvae (*Cephus cinctus*) treated with three species of EPNs (*Heterorhabditis indica*, *Steinernema feltiae*, and *Steinernema kraussei*), 2 days and 3 days after exposure.

IJs /larva	*S*. *feltiae*		*H*. *indica*		*S*. *kraussei*	
	Day 2	Day 3	Day 2	Day 3	Day 2	Day 3
0	0 ± 0.0	0 ± 0.0	0 ± 0.0	0 ± 0.0	0 ± 0.0	0 ± 0.0
50	60 ± 21.9	80 ± 17.9	100 ± 0.0	100 ± 0.0	20 ± 17.9	40 ± 21.9
100	40 ± 21.9	60 ± 21.9	100 ± 0.0	100 ± 0.0	40 ± 21.9	60 ± 21.9
200	20 ± 17.9	100 ± 0.0	100 ± 0.0	100 ± 0.0	40 ± 21.9	80 ± 17.9
500	80 ± 17.9	100 ± 0.0	100 ± 0.0	100 ± 0.0	40 ± 21.9	60 ± 21.9

Distilled water and thirteen different chemical carrier solutions were prepared according to Table 1 and stored at 4°C. *H*. *bacteriophora*, *S*. *feltiae*, *and S*. *riobrave* were obtained from a commercial supplier (Sierra Biological, Pioneer CA) and stored at 4°C. EPNs were allowed to equilibrate to room temperature (22°C) before being added to 4 ml of each carrier solution. Solution volumes were adjusted to achieve concentrations of approximately 2000 IJ/ml.

Soil was collected from an onsite field plot, rocks and other debris were removed manually, and distilled water was added to bring the soil moisture level to ~30%. The soil was sterilized at 125°C for 45 mins in an autoclave. Previously collected wheat stubble, which housed diapausing WSS, was removed from cold storage (8°C) and 15–20 individual stems were inserted into 473 ml deli cups containing approximately 150 ml of the moist autoclaved soil. Using disposable pipettes, solutions containing EPNs were mixed thoroughly and applied to the wheat stems by placing a single droplet (~20 ul) on top of the stem’s plug. To determine if WSS were previously infected by naturally occurring EPNs, subsets of stems were treated with distilled water containing no EPNs (negative control). The order of treatment applications was randomized and treated stems were incubated at 25°C in a growth chamber (14:10 L/D, 50% RH) for 7 days.

Following incubation, stems were sliced open with a scalpel along the long axis and larvae or pupae were carefully removed with forceps or a dissecting needle. Both larvae and pupae were found because the insects were slowly developing during the four months in cold storage. Individuals that appeared infected with EPNs were dissected under a stereomicroscope to confirm the presence of EPNs; individuals that appeared healthy were placed in small 59 ml portion cups and monitored for seven days for latent signs of infection. WSS percent mortalities were calculated from groups of 15–20 stems contained in each deli cup. The assay was subsequently repeated two more times on different dates (N = 3). Mortality was assessed for a total of 1173 larvae and 288 pupae (15–20 stems × 14 carrier solutions × 3 EPNs × 3 repetitions). Percent mortalities from each repetition were averaged within treatments (carrier solutions × EPNs) to obtain mean percent mortality values.

### Field trials of EPNs with carrier solutions

The previous experiment demonstrated that Penterra, Silwet L-77, Sunspray 11N, and Syl-Tac performed better at allowing EPNs to enter stems compared to all other adjuvants, thus, these four carrier solutions, as well as, Barricade and distilled water were selected for field tests. Although water and Barricade were not top performers in the laboratory assay, they were included in our field tests because EPNs are typically mixed with water for spray applications, and Barricade has been used successfully to increase the efficiency of EPNs against above-ground insects [31, 27, 28]. All three species of EPNs were tested with the six different carrier solutions at three field locations (3 × 6 × 3 Randomized Complete Block design)–untreated stems served as negative controls to determine if any WSS were infected with indigenous EPNs. In early May 2016, field plots were established in three previously harvested (fall 2015) Judee winter wheat fields; two locations (Bjelland Farm and Johnson Farm) in Pondera county MT (N48°10.567’ W111°32.872’; N48°11.397’ W111°25.843’) and one location (Schuler Farm) in Teton county MT (N47°52.360’ W111°40.324’). Permission to conduct field trials was granted by local private landowners as mentioned above. Soil type at each location consisted of silty clay loam. Field plots were 1 m^2^ and contained 3–4 rows of wheat stubble. The corners of the plots were marked with orange painted wooden stakes. To minimize variation in WSS densities [21], plots were arranged linearly approximately equal distances from the edges of the fields. Individual plots were spaced ~8.0 m apart to avoid effects from overspray or migration of EPNs and plot order was randomized at each location [37].

Carrier solutions were prepared fresh and EPNs added at a concentration of 1000 IJs/ml–the lower EPN concentration more closely simulated real-life application conditions. After adding EPNs, treatment solutions were kept at 8°C prior to transporting to the field sites in order to conserve the EPN’s energy reserves and minimize their temperature related stress response. In the field, 100 ml of the treatment solutions were added to 3.79 L pressurized hand sprayers (H.D. Hudson Manufacturing Company Chicago, IL)–this volume also more closely simulated real-life application conditions of. All sprayers were pressurized with 25 pumps of the handle (>100 psi) which provided enough pressure to apply the more viscous 1.0% Barricade but still below 200 psi which can cause mortality to EPNs [38]. To standardize the spray rate and spray pattern, a single spray nozzle was interchanged between sprayers for all treatments. The nozzle was adjusted to provide an even cone-shaped spray pattern ~15 cm wide at a height of 15–20 cm. Between each treatment, the nozzle was rinsed for 3 sec each with soapy water, then tap water, which thoroughly removed any remaining solution from the previous treatment. Treatment solutions were applied evenly to each plot by holding the tip of the nozzle ~15–20 cm above the soil level and moving the nozzle back and forth in a sweeping motion until the liquid was exhausted. To minimize UV exposure and high daytime temperatures, treatment solutions were applied just before sunset. Average air temperatures during treatment applications were 17.2°C, 15.2°C, and 17.2°C at the Bjelland, Johnson, and Shuler Farms, respectively. Average daily air temperatures and daily RH for the five day treatment periods were 10.6°C; 79% RH, 10.0°C; 78% RH, and 12.2°C; 81% RH at the Bjelland, Johnson, and Shuler Farms, respectively.

Five days after treatment, five clumps of wheat stubble were randomly collected from each plot and placed in clean zip-lock bags during transport back to the laboratory. Rainy conditions (0.85 cm / day, May 20–22) during collecting caused the wheat clumps to be soggy, thus wheat clumps were allowed to dry for ~24 hrs before separating. Stems containing diapausing larvae or pupae were removed from the wheat clump, cleaned of dirt and debris, and placed in 473 ml plastic deli containers. Stems were stored at 8°C until they could be assayed for the presence of EPNs (<5 days). Twenty stems (various lengths) from each plot were randomly selected and carefully sliced open with a scalpel to expose the larvae (248 total) or pupae (827 total). All larvae and pupae were assayed for mortality. Dead larvae or pupae were dissected under a stereomicroscope to look the presence of EPNs; individuals that appeared healthy were placed in small 59 ml portion cups and observed for 7 days for latent signs of infection. WSS percent mortality was calculated for each treatment plot, at each location, and percent mortalities were averaged across locations (N = 3) to obtain mean percent mortality values for all treatments (carrier solutions × EPNs).

### Data analysis

Many factors can cause mortality in WSS populations (*e*.*g*. environment conditions, parasitoids, fungi, pathogens, etc.). Therefore, treatment percent mortalities from both laboratory and field tests were adjusted using the Schneider-Orelli formula to correct for percent mortalities found in control samples [39]. Initial two-way analysis of variance (ANOVA) showed no significant percent mortality differences in larvae vs. pupae (P = 0.12), thus larval and pupal mortalities were pooled among treatments (EPNs × solutions).

For the laboratory experiment, treatment (EPNs × solutions) percent mortalities from each repetition were treated as independent samples (N = 3). Two-way ANOVA was used compare differences in WSS percent mortalities among treatments. The ANOVA model (R^2^ = 0.47, P<0.0001) for the laboratory experiment included “*EPN species*” and “*carrier solution*” as predictor variables. The “*EPN* × *solution*” interaction term was not significant (P = 0.552) and was removed from the model. Post-hoc multiple comparisons (Dunnett’s test, α = 0.05) were used to determine differences in WSS mortality when stems were treated with EPNs mixed with chemical carrier solutions vs. EPNs mixed with distilled H_2_O (control). Tukey’s Honest Significant Difference (α = 0.05) was used to test for WSS mortality differences among the three EPNs.

For the field experiment, treatment (EPNs × solutions) percent mortalities from each location were treated as independent samples (N = 3). Two-way ANOVA was used to compare differences in WSS percent mortalities among treatments. The ANOVA model (R^2^ = 0.59, P<0.0001) included “*farm*”, “*EPN species*”, and “*EPN* × *farm*” interaction term as predictor variables–“*carrier solution*” was not significant. Post-hoc multiple comparisons (Tukey’s HSD, α = 0.05) were used to test for differences in WSS percent mortality for all three predictor variables. All analyses were carried out in JMP v. 12 (SAS Institute, Cary, NC).

## Results

### EPN infection assay

This test confirmed that three species of EPNs have the ability to infect and kill WSS larvae. *H*. *indica* proved to be the most virulent species because WSS mortality was 100% after day 2 for all concentrations of IJs (Table 3). High concentrations of *S*. *feltiae* (200, 500 IJ/larva) also produced 100% mortality by day 3. The highest mortality achieved by *S*. *kraussei* was 60%, making it the least virulent of the EPNs tested. EPN related differences in WSS mortality suggest that WSS is more susceptible to infection and death from *H*. *indica* and *S*. *feltiae*, compared to *S*. *kraussei*.

### Adjuvant absorbance assay

Water alone does not readily absorb into plugs formed by the WSS, therefore, we tested a variety of commercially available adjuvants including: surfactants, wetting agents, oils, and a humectant (Barricade) for their ability to increase absorption. Artificial plugs released into distilled water required more than 5 min to become completely saturated. Plugs would float on the surface of the water for a considerable amount of time (~2–3 min) before the water would begin to absorb–affirming the hydrophobic nature of the plug material. The amount of time required for the plugs to be completely saturated in the different solutions was variable (Table 2); however, saturation occurred most rapidly in R-11 (4.2 ± 0.03 sec). Plugs were also saturated quickly in Syl-Tac and Adigor (6.5 ± 0.85 and 12.4 ± 3.58 sec, respectively). This result indicates that chemical additives would allow EPN suspensions to absorb into the plug >50× more rapidly than EPN suspensions made with water alone.

### Laboratory assay of EPNs with carrier solutions

**Fig 1:**
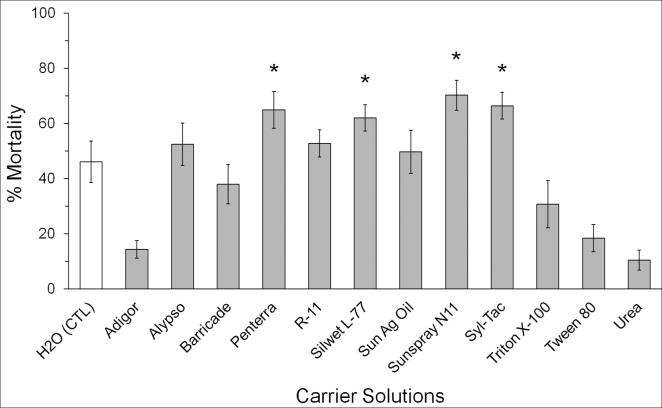
Mortality of wheat stem sawfly (*Cephus cinctus*) from wheat stubble treated with three species of EPNs (*Heterorhabditis bacteriophora*, *Steinernema feltiae*, and *Steinernema riobrave*) combined with different carrier solutions. Percent mortalities were pooled across EPN species and bars represent average percent mortality (mean ± SEM) for each treatment solution (N = 9). Asterisks indicate significant differences in percent mortality (Dunnett’s test, α = 0.05) compared to controls (H_2_O).

This assay demonstrated that certain chemical additives improved the ability of EPNs to penetrate the plug and infect the residing WSS larvae or pupae. On average, WSS mortality was significantly higher (F = 9.49, df = 12, P<0.0001) when EPNs were mixed with Penterra (P = 0.015), Silwet L-77 (P = 0.043), Sunspray 11N (P = 0.002), or Syl-Tac (P = 0.008), compared to EPNs mixed with distilled water (Fig 1)–two of these solutions (Silwet L-77, and Syl-Tac) contained silicone-based polymers. There were also EPN related differences in WSS mortality (F = 6.69, df = 2, P = 0.002). On average *S*. *riobrave* and *S*. *feltiae* inflicted 50.5% and 47.1% mortality, respectively–significantly higher (P = 0.002, P = 0.019) than 35.0% mortality from *H*. *bacteriophora*. This result indicates that *S*. *riobrave* and *S*. *feltiae* are better at penetrating the plug and infecting diapausing WSS than *H*. *bacteriophora*.

### Field trials of EPNs with carrier solutions

**Fig 2:**
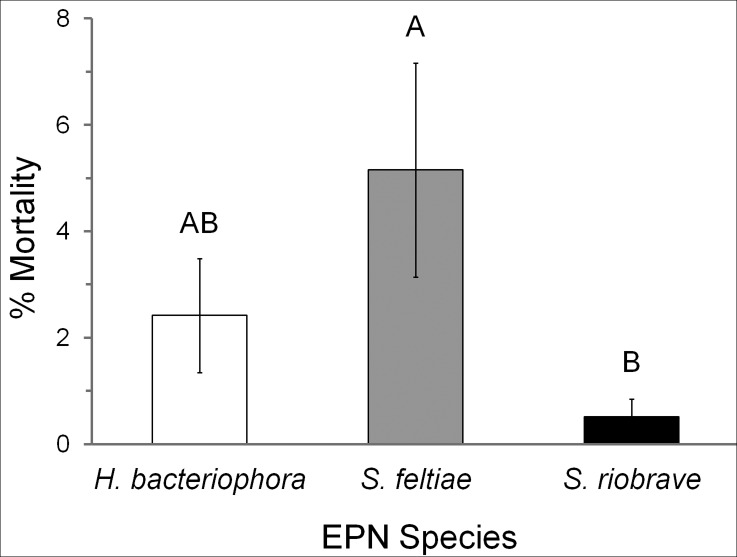
Mortality of wheat stem sawfly (*Cephus cinctus*) from field wheat stubble treated with three species of EPNs (*Heterorhabditis bacteriophora*, *Steinernema feltiae*, and *Steinernema riobrave*). Percent mortalities were pooled across EPN species and bars represent average percent mortality (mean ± SEM) for each species (N = 18). Different letters indicate significant differences in percent mortality (Tukey’s HSD, α = 0.05).

In the field, solutions containing *S*. *feltiae* and 0.1% Penterra increased WSS mortality up to 29% in harvested winter wheat stubble. On average, solutions containing *S*. *feltiae* increased WSS mortality (5.1%) more than *H*. *bacteriophora* or *S*. *riobrave* (F = 6.87, df = 2, P = 0.003; Fig 2), and *S*. *feltiae* combined with Penterra, resulted in the highest average mortality (9.78%; Table 4). However, *S*. *feltiae’s* effectiveness varied extensively across the three locations (Table 5); hence, location also had a significant effect on WSS mortality (F = 14.71, df = 2, P<0.0001). WSS percent mortality was higher at the Schuler farm compared to the other locations (P<0.0001). Multiple comparisons of the *EPN* × *farm* interaction showed that *S*. *feltiae* was more effective at the Schuler farm (15.5%) compared to all other EPN-location combinations (F = 9.95, df = 4, P<0.0001); no significant location-related mortality differences were found for *H*. *bacteriophora* or *S*. *riobrave*. These results indicate that spraying winter wheat stubble with solutions containing *S*. *feltiae* mixed with 0.1% Penterra may result in a significant decrease in the number of developing WSS larvae and pupae.

**Table 4:** Average (mean ± SE), minimum, and maximum percent field mortality (N = 3) of wheat stem sawfly (*Cephus cinctus*) from wheat stubble treated with three species of EPNs (*Heterorhabditis bacteriophora*, *Steinernema feltiae*, and *Steinernema riobrave*) combined with different carrier solutions.

Adjuvant	EPN species	% Mortality		
		Average	Minimum	Maximum
	*H*. *bacteriophora*	0.0 ± 0.0	0.0	0.0
Distilled H_2_0	*S*. *feltiae*	4.2 ± 4.2	0.0	12.7
	*S*. *riobrave*	0.0 ± 0.0	0.0	0.0
	*H*. *bacteriophora*	3.9 ± 3.9	0.0	11.7
Barricade	*S*. *feltiae*	4.2 ± 4.2	0.0	12.7
	*S*. *riobrave*	0.0 ± 0.0	0.0	0.0
	*H*. *bacteriophora*	3.9 ± 3.9	0.0	11.7
Penterra	*S*. *feltiae*	9.7 ± 9.7	0.0	29.1
	*S*. *riobrave*	0.0 ± 0.0	0.0	0.0
	*H*. *bacteriophora*	0.0 ± 0.0	0.0	0.0
Silwet L-77	*S*. *feltiae*	6.1 ± 6.1	0.0	18.2
	*S*. *riobrave*	2.5 ± 1.6	0.0	5.6
	*H*. *bacteriophora*	4.1 ± 3.5	0.0	11.1
Sunspray 11N	*S*. *feltiae*	4.2 ± 4.2	0.0	12.7
	*S*. *riobrave*	0.6 ± 0.6	0.0	1.8
	*H*. *bacteriophora*	2.6 ± 2.6	0.0	7.7
Syl-Tac	*S*. *feltiae*	2.4 ± 2.4	0.0	7.3
	*S*. *riobrave*	0.0 ± 0.0	0.0	0.0

**Table 5:** Average (mean ± SE), minimum, and maximum percent field mortality (N = 3) of wheat stem sawfly (*Cephus cinctus*) from wheat stubble treated with three species of EPNs (*Heterorhabditis bacteriophora*, *Steinernema feltiae*, and *Steinernema riobrave*) at three different locations.

Farm	EPN species	% Mortality		
		Average	Minimum	Maximum
	*H*. *bacteriophora*	1.3 ± 1.3	0.0	7.7
Bjelland	*S*. *feltiae*	0.0 ± 0.0	0.0	0.0
	*S*. *riobrave*	0.3 ± 0.3	0.0	1.9
	*H*. *bacteriophora*	1.9 ± 1.9	0.0	11.1
Johnson	*S*. *feltiae*	0.0 ± 0.0	0.0	0.0
	*S*. *riobrave*	0.9 ± 0.9	0.0	5.6
	*H*. *bacteriophora*	4.1 ± 2.4	0.0	11.7
Schuler	*S*. *feltiae*	15.5 ± 3.1	7.3	29.1
	*S*. *riobrave*	0.3 ± 0.3	0.0	1.8

## Discussion

The WSS, *Cephus cinctus*, has been a major pest of winter wheat and barley in the northern Great Plains for more than 100 years [12]. To date, cultural practices and parasitoids have not been sufficient to broadly control WSS [21, 5]. Presently, biological control offers the best hope for economical and sustainable management of WSS. To our knowledge this is the first study to confirm that *C*. *cinctus* larvae and pupae are susceptible to infection by EPNs, and that EPNs can penetrate the wheat stubble to infect diapausing insects–suggesting a novel control method for this persistent pest. We compared percent mortalities of diapausing WSS larvae and pupae after applying EPNs mixed with different carrier solutions (including distilled water) to winter wheat stubble. We found that EPNs can inflict 60–100% mortality on WSS when EPNs come into contact with the insect. We also showed that adding different adjuvants (Penterra, Silwet L-77, Sunspray 11N, or Syl-Tac) to solutions containing EPNs increased the ability of three species of EPNs (*H*. *bacteriophora*, *S*. *feltiae*, and *S*. *riobrave*) to get through the plug and infect the diapausing WSS residing inside the stem lumen. On average, field treatments resulted in 2.7% percent mortality of WSS. However, WSS mortality was highly variable among different treatments and field sites; at one field site, 29.1% WSS mortality was achieved by adding Penterra to a spray solution containing *S*. *feltiae*. Our results are encouraging because they indicate that adding certain adjuvants to sprays containing EPNs can facilitate incursion of EPNs into the stems, resulting in a significant increase in WSS mortality in post-harvest winter wheat fields.

The six EPNs used in our initial laboratory experiments consisted of species that used either cruising (*H*. *bacteriophora*, *S*. *glaseri*, *S*. *kraussei*), ambushing (*S*. *carpocapsae*), or intermediate (*S*. *feltiae*, *S*. *riobrave*) foraging strategies. Because EPNs must find their way into the wheat stem to infect the WSS, perhaps it is not surprising that the three species that produced the highest levels of mortality in our laboratory tests (*H*. *bacteriophora*, *S*. *feltiae*, and *S*. *riobrave*) exhibited either cruising or intermediate foraging strategies (i.e. more mobile species). *S*. *glaseri*, and *S*. *kraussei* are also mobile EPNs [22], however, treatment solutions containing both species produced low WSS mortality (0.0–15.4% and 5.6–16.7%, respectively) compared to treatments containing *H*. *bacteriophora*, *S*. *feltiae*, and *S*. *riobrave* (22.2–64.7%, 23.5–33.1%, and 14.8–42.1%, respectively). IJs of *S*. *glaseri* are very large (8× volume) compared to the IJs of the other species of EPNs tested, thus they may have had greater difficulty finding their way through the plug. It is unclear why treatments containing *S*. *kraussei* produced low WSS mortality; however, this result illustrates the importance of testing the efficiency of various EPN species against new insect pests.

Under laboratory conditions, mortality of WSS treated with carrier solutions containing *S*. *feltiae* and *S*. *riobrave* were about equal (~50%); however, under field conditions, *S*. *feltiae* achieved significantly higher levels of mortality than *S*. *riobrave* (P = 0.0001). Since northwest Montana is semi-arid, the underperformance of *S*. *riobrave* was a surprising result because *S*. *riobrave* is reported to be tolerant of dry conditions. Low WSS mortalities that resulted from treatments containing *S*. *riobrave* might be explained by unfavorable soil conditions at the field sites. The soil in northwest Montana contains high clay content. The foraging efficiency of *S*. *riobrave* declines in clay loam soil [40]. Additionally, low nocturnal temperatures could have negatively affected *S*. *riobrave’s* infectivity. *S*. *riobrave* is active and infective at temperatures ranging from 15–35°C, while *S*. *feltiae* is active and infective at temperatures ranging from 10–30°C [22]–which suggests that *S*. *feltiae* is better adapted to lower temperatures than *S*. *riobrave*. We applied our field treatments right before sundown, when the average air temperature for the three sites was 16.5°C, which is above the minimum temperature threshold for both species. However, nocturnal air temperatures at the sites dropped below 10°C, which could have reduced the foraging activity of *S*. *riobrave* more than *S*. *feltiae*. Lastly, wet conditions in the fields due to rainfall (2.5 cm total) during the experiment may have also contributed to the low performance of *S*. *riobrave*. *S*. *riobrave* is adapted to dry soil conditions; hence, too much moisture could negatively impact *S*. *riobrave’s* foraging. In contrast, high soil moisture has been shown to increase the foraging efficiency of *S*. *feltiae* [41].

Our results are consistent with other studies showing that EPN efficiency against above-ground pests can be enhanced when combined with adjuvants or humectants. Combining TX7719 with Blankophor BBH was found to increase EPN persistence and efficacy against *Plutella xylostella* L. on watercress leaves [30]. Adding Silwet L-77, SBPI or Addit to solutions containing EPNs resulted in a 2-fold increase in EPN deposition [42], and adding 0.3% surfactant resulted in ~38% reduction in the amount of time required for *S*. *carpocapsae* to cause 50% mortality in *P*. *xylostella* [33]. In contrast, a few studies have shown that certain adjuvants can have negative effects on EPN survival and mobility [29]. Some adjuvants, such as alcohol ethoxylates and alkyl polysaccharides were reported to cause temporary immobilization of EPNs [42, 43]. We evaluated the viability of *H*. *bacteriophora*, *S*. *feltiae*, and *S*. *riobrave* in all of the carrier solutions over 48 hrs, but did not observe any considerable decreases in EPN activity or survival (data not shown)–indicating that the carrier solutions that we tested did not cause serious harm to the EPNs.

Previous studies have also shown that adding Barricade (humectant) to EPN spray mixtures improves the efficiency of EPNs used against above ground insect pests. EPNs are vulnerable to desiccation under direct sunlight or low humidity conditions (e.g. northern Great Plains). Humectants provide EPNs with moisture and protection from UV, thus prolonging their survival and host finding capability when applied to the surface of foliage [31]. A recent study showed that canola (*Brassica napus* L.), under high crucifer flea beetle (*Phyllotreta cruciferae* Goeze) feeding pressure, produced the highest yields when sprayed with *S*. *feltiae* added to 1% Barricade [44]. EPN treatments containing 0.25% or 0.5% Barricade resulted in higher mortality of *Spodoptera litura* F. larvae (66.0% and 61.5%, respectively) compared to EPNs mixed with tap water (29.5%); Barricade treatments also increased the mortality of *P*. *xylostella* larvae [32]. Moreover, adding Barricade to EPN treatments has been used successfully to manage stem-boring pests [31, 27, 28]. Despite the effectiveness of EPN/Barricade formulations at reducing other insect pests, our results showed a marginal positive effect from treatments that included Barricade. Our field test showed that 1% Barricade treatments with *H*. *bacteriophora* resulted in WSS mortality on par with Penterra and Sunspray 11N, and 1% Barricade treatments with *S*. *feltiae* result in lower WSS mortality levels than treatments containing Penterra or Silwet L-77 (Table 5). The performance of EPN/Barricade treatments in our field trials was consistent with the results we obtained from our laboratory test. This indicates that our laboratory test offered reasonable predictions as to how different treatments will perform in field situations.

Recently it has been reported that EPN solutions containing 1% Barricade resulted in higher insect mortality than other formulations [31]. We also chose to use 1% Barricade solution because the 1% solution resulted in more even spray coverage and was less likely to clog the spray nozzles than solutions >1%. 1% Barricade provided better protection to the EPNs than the other carrier solutions because it was considerably more viscous and retained it’s gelatinous and adhesive qualities. Adding less Barricade to spray mixtures would have economic benefits for growers as well. Furthermore, adding titanium dioxide to outdoor EPN treatments resulted in a ~10× increase in insect mortality compared to treatments without titanium dioxide [31]. This result indicates that the UV protective properties of titanium dioxide improved the EPN’s survival and host killing efficiency. Adding titanium dioxide to EPN treatments against WSS might be beneficial to EPN survival and efficacy and would be an interesting topic for further research.

One factor that might have contributed to the EPN’s success at penetrating the stem lumen in our field trial was applying our field treatments in late spring (May 17th and 18th, 2016) vs. fall the previous year (2015). Wheat stubble harvested in fall will have decomposed to some degree by the following spring. Therefore, applying treatments when the wheat stem tissue, and possibly the plug, has had a chance to breakdown could have facilitated the EPN’s ingress into the stems. Our results are consistent with a previous study that showed a significant reduction in the number of WSS infested wheat stems collected from plots treated with five species of EPNs. This study also showed that the number of WSS larvae present in wheat stubble was lower in plots treated with *S*. *feltiae* compared to *H*. *bacteriophora* [34]. Our results are also, consistent with studies showing that EPNs can cause high levels of mortality in other stem-boring insect pests. *Steinernema* and *Heterorhabditis* spp. are reported to infect banana weevil larvae (*Cosmopolites sordidus* Germar) inside the plant stems [25]. Field applications of *S*. *riobrave* and *S*. *feltiae* were shown to provide the same control level of the squash vine borer (*Melittia cucurbitae* Harris) as the insecticide Endosulfan^®^ [23]. Another study reported that a combination of host plant cultivar and treatments with *H*. *indica* produced effective management of the rice stem borer (*Maliarpha separatella* Ragonot) [24]. These studies (including our own) indicate that EPNs can be effective for controlling some stem-boring insects. Hence, EPNs should be seriously considered when exploring biological control options for stem-boring pests.

EPN treatments will not help growers recover losses due to WSS damage to previously harvested crops, but continual applications of EPNs to post-harvest wheat stubble may reduce crop losses in subsequent years. More studies are needed to optimize the methods, and large scale trails are required to confirm the efficacy of this system. Future experiments are also required to determine if consistent yearly treatments using EPNs will result in a significant decline in WSS populations over time. Additionally, there is evidence that EPN infectivity may be enhanced when they are combined with other biorational insecticides such as Spinosad® [45], but this synergy has not been tested against WSS. EPNs can also be added to other spray mixtures (e.g. fertilizers, herbicides) so growers can save time, water, and expense by not having to apply additional field treatments [46]–conserving water is an important consideration for Montana growers because water reserves are budgeted in the region. Supplemental experiments are also needed to ensure that chemical fertilizers, herbicides or insecticides used in Montana will not harm EPNs. Because 40–95% of WSS larvae are found along the edges of fields [47], EPN treatments may only need to be applied to field edges–further reducing application times and costs. Ultimately, cost/benefit analysis will determine if this method is economical and sustainable for wheat and barley growers in the Golden Triangle.

## Supporting Information

S1 Data Data File WSS vs. EPNs.xlsx (XLSX)
